# Supplementary material for: Systemic nicotinamide mononucleotide administration to mitigate post-cardiac arrest brain injury in mice
Source: PLoS One. 2025 Oct 21;20(10):e0334608. doi: 10.1371/journal.pone.0334608 (PMC12539731; doi:10.1371/journal.pone.0334608)
Supplement: S1 Table — (DOCX) [file pone.0334608.s001.docx]

**S1 Table. Baseline characteristics, resuscitation details, and vital signs of individual mice in experiment 2.**

**A. Baseline characteristics and resuscitation details**

| **Group** | **Mouse** | **Age (w)** | **Weight (g)** | **CPR time (min)** | **Epinephrine dose (μg)** |
| --- | --- | --- | --- | --- | --- |
| Control | 1 | 15 | 27.8 | 1.5 | 6.00 |
|  | 2 | 14 | 25.9 | 1.3 | 6.00 |
|  | 3 | 13 | 23.7 | 1.2 | 6.00 |
|  | 4 | 13 | 24.9 | 1.3 | 8.23 |
|  | 5 | 13 | 24.3 | 1.8 | 8.88 |
|  | 6 | 12 | 26.1 | 2.0 | 8.21 |
|  | 7 | 12 | 27.2 | 1.6 | 7.16 |
|  | 8 | 12 | 25.5 | 1.4 | 6.00 |
|  | 9 | 13 | 24.7 | 2.8 | 7.54 |
|  | 10 | 13 | 24.4 | 1.6 | 7.13 |
|  | 11 | 13 | 26.5 | 1.7 | 8.98 |
|  | 12 | 14 | 26.8 | 1.6 | 6.00 |
|  | 13 | 14 | 29.1 | 2.0 | 7.81 |
|  | 14 | 11 | 26.2 | 1.8 | 6.00 |
|  | 15 | 11 | 24.6 | 1.5 | 7.08 |
|  | 16 | 11 | 24.0 | 2.3 | 11.81 |
|  | 17 | 11 | 24.2 | 1.6 | 7.20 |
|  | 18 | 11 | 23.5 | 2.9 | 6.00 |
| NMN | 1 | 13 | 26.5 | 1.8 | 6.65 |
|  | 2 | 13 | 23.8 | 3.1 | 11.07 |
|  | 3 | 13 | 28.0 | 1.6 | 7.65 |
|  | 4 | 12 | 25.5 | 2.0 | 8.00 |
|  | 5 | 12 | 24.6 | 1.6 | 6.00 |
|  | 6 | 13 | 25.7 | 1.9 | 6.00 |
|  | 7 | 13 | 27.5 | 1.8 | 6.00 |
|  | 8 | 13 | 26.8 | 2.1 | 6.00 |
|  | 9 | 13 | 25.7 | 1.3 | 7.36 |
|  | 10 | 13 | 27.9 | 2.2 | 7.09 |
|  | 11 | 14 | 26.3 | 2.0 | 6.00 |
|  | 12 | 14 | 27.4 | 2.4 | 7.77 |
|  | 13 | 13 | 26.1 | 1.7 | 6.00 |
|  | 14 | 11 | 24.4 | 2.1 | 6.00 |
|  | 15 | 11 | 24.5 | 1.8 | 6.00 |
|  | 16 | 11 | 23.3 | 3.2 | 8.92 |
|  | 17 | 11 | 24.3 | 1.5 | 6.00 |
|  | 18 | 11 | 23.9 | 2.6 | 9.26 |

**B. Vital signs at baseline and 30 min after ROSC.**

| **Group** | **Mouse** | **Baseline variables** | | | **30 min after ROSC** | | |
| --- | --- | --- | --- | --- | --- | --- | --- |
|  |  | **HR (/min)** | **MAP (mmHg)** | **BT (℃)** | **HR (/min)** | **MAP (mmHg)** | **BT (℃)** |
| Control | 1 | 404 | 94 | 35.8 | 665 | 112 | 36.5 |
|  | 2 | 407 | 92 | 35.9 | 616 | 105 | 36.2 |
|  | 3 | 403 | 83 | 36.3 | 574 | 100 | 36.7 |
|  | 4 | 405 | 90 | 35.7 | 553 | 119 | 37.0 |
|  | 5 | 418 | 85 | 35.7 |  |  |  |
|  | 6 | 434 | 93 | 35.6 | 674 | 119 | 35.5 |
|  | 7 | 358 | 80 | 35.9 | 585 | 126 | 36.1 |
|  | 8 | 331 | 106 | 36.0 | 589 | 119 | 36.4 |
|  | 9 | 356 | 86 | 36.6 | 606 | 92 | 36.6 |
|  | 10 | 368 | 82 | 36.7 | 586 | 104 | 36.7 |
|  | 11 | 477 | 82 | 37.3 | 621 | 87 | 37.0 |
|  | 12 | 253 | 80 | 35.8 | 606 | 105 | 35.9 |
|  | 13 | 596 | 114 | 35.6 | 641 | 107 | 36.9 |
|  | 14 | 518 | 92 | 33.0 | 624 | 119 | 35.7 |
|  | 15 | 414 | 89 | 36.9 | 317 | 95 | 36.2 |
|  | 16 | 425 | 102 | 36.8 | 568 | 105 | 36.4 |
|  | 17 | 301 | 88 | 35.3 |  |  |  |
|  | 18 | 357 | 80 | 35.8 | 603 | 112 | 36.4 |
| NMN | 1 | 376 | 88 | 35.9 | 612 | 114 | 36 |
|  | 2 | 444 | 97 | 34.7 | 446 | 126 | 35.9 |
|  | 3 | 479 | 92 | 37.6 | 601 | 115 | 36.7 |
|  | 4 | 301 | 79 | 36.2 | 666 | 111 | 36.9 |
|  | 5 | 446 | 95 | 34.6 | 572 | 119 | 36.3 |
|  | 6 | 349 | 109 | 35.9 | 602 | 115 | 36.8 |
|  | 7 | 405 | 91 | 36.1 |  |  |  |
|  | 8 | 572 | 98 | 34.2 | 592 | 111 | 36.1 |
|  | 9 | 408 | 78 | 35.6 | 596 | 97 | 36.6 |
|  | 10 | 547 | 102 | 35.3 | 561 | 110 | 35.8 |
|  | 11 | 238 | 84 | 35.7 | 600 | 125 | 35.8 |
|  | 12 | 480 | 99 | 34.6 | 569 | 115 | 35.9 |
|  | 13 | 375 | 89 | 36.4 |  |  |  |
|  | 14 | 293 | 98 | 35.5 | 598 | 108 | 36 |
|  | 15 | 398 | 76 | 34.4 | 618 | 113 | 35.3 |
|  | 16 | 346 | 89 | 35.6 | 598 | 111 | 36.7 |
|  | 17 | 301 | 101 | 35.3 | 563 | 105 | 36.2 |
|  | 18 | 354 | 94 | 35.4 | 562 | 125 | 36.1 |
