# Supplementary material for: Systemic nicotinamide mononucleotide administration to mitigate post-cardiac arrest brain injury in mice
Source: PLoS One. 2025 Oct 21;20(10):e0334608. doi: 10.1371/journal.pone.0334608 (PMC12539731; doi:10.1371/journal.pone.0334608)
Supplement: S2 Table — (DOCX) [file pone.0334608.s002.docx]

**S2 Table. Neurological function score and survival time of individual mice in experiment 2.**

| **Group** | **Mouse** | **Neurological function score** | | **Survival time** |
| --- | --- | --- | --- | --- |
|  |  | **Day 1** | **Day 2** | **(days)** |
| Control | 1 | 10 | 10 | 7 |
|  | 2 | 10 | 8 | 3 |
|  | 3 | 7 | 8 | 6 |
|  | 4 | 11 | 0 | 1 |
|  | 5 | 8 | 0 | 1 |
|  | 6 | 7 | 4 | 2 |
|  | 7 | 7 | 7 | 3 |
|  | 8 | 12 | 12 | 7 |
|  | 9 | 0 | 0 | 0 |
|  | 10 | 5 | 4 | 2 |
|  | 11 | 0 | 0 | 0 |
|  | 12 | 12 | 12 | 7 |
|  | 13 | 6 | 0 | 1 |
|  | 14 | 12 | 12 | 7 |
|  | 15 | 6 | 0 | 1 |
|  | 16 | 0 | 0 | 0 |
|  | 17 | 7 | 4 | 4 |
|  | 18 | 2 | 4 | 2 |
| NMN | 1 | 0 | 0 | 0 |
|  | 2 | 7 | 9 | 4 |
|  | 3 | 0 | 0 | 0 |
|  | 4 | 12 | 12 | 7 |
|  | 5 | 12 | 12 | 7 |
|  | 6 | 11 | 7 | 2 |
|  | 7 | 10 | 10 | 7 |
|  | 8 | 12 | 12 | 7 |
|  | 9 | 5 | 0 | 1 |
|  | 10 | 4 | 9 | 7 |
|  | 11 | 12 | 12 | 7 |
|  | 12 | 6 | 7 | 7 |
|  | 13 | 12 | 12 | 7 |
|  | 14 | 12 | 12 | 7 |
|  | 15 | 12 | 12 | 7 |
|  | 16 | 5 | 0 | 1 |
|  | 17 | 12 | 12 | 7 |
|  | 18 | 4 | 0 | 1 |
