# Supplementary material for: Systemic nicotinamide mononucleotide administration to mitigate post-cardiac arrest brain injury in mice
Source: PLoS One. 2025 Oct 21;20(10):e0334608. doi: 10.1371/journal.pone.0334608 (PMC12539731; doi:10.1371/journal.pone.0334608)
Supplement: S3 Table — (DOCX) [file pone.0334608.s003.docx]

**S3 Table. Percentages of FJC-positive areas for individual mice in experiment 3.**

| **Time post-CA** | **Group** | **Mouse** | **FJC-positive areas (%)** |
| --- | --- | --- | --- |
| 24 h | Control | 1 | 1.30 |
|  |  | 2 | 1.04 |
|  |  | 3 | 1.45 |
|  |  | 4 | 1.87 |
|  | NMN | 1 | 0.85 |
|  |  | 2 | 1.11 |
|  |  | 3 | 1.47 |
|  |  | 4 | 0.18 |
| 48 h | Control | 1 | 4.77 |
|  |  | 2 | 7.32 |
|  |  | 3 | 6.64 |
|  |  | 4 | 3.88 |
|  |  | 5 | 2.23 |
|  | NMN | 1 | 2.26 |
|  |  | 2 | 2.54 |
|  |  | 3 | 3.42 |
|  |  | 4 | 0.97 |
|  |  | 5 | 0.48 |
