# Supplementary material for: Systemic nicotinamide mononucleotide administration to mitigate post-cardiac arrest brain injury in mice
Source: PLoS One. 2025 Oct 21;20(10):e0334608. doi: 10.1371/journal.pone.0334608 (PMC12539731; doi:10.1371/journal.pone.0334608)
Supplement: S4 Table — (DOCX) [file pone.0334608.s004.docx]

**S4 Table. qPCR cycle threshold values and protein levels of individual mice in experiment 4.**

**A. *Sirt3***

| **Group** | **Mouse** | **Ct** | | **Δ Ct** | **ΔΔ Ct** | **Fold Change** |
| --- | --- | --- | --- | --- | --- | --- |
|  |  | ***Sirt3*** | ***Gapdh*** |  |  |  |
| Control | 1 | 21.246 | 15.673 | 5.573 | 0.228 | 0.85 |
|  | 2 | 21.271 | 15.830 | 5.442 | 0.097 | 0.93 |
|  | 3 | 20.985 | 15.966 | 5.019 | -0.326 | 1.25 |
| NMN | 1 | 20.500 | 15.706 | 4.794 | -0.551 | 1.46 |
|  | 2 | 20.178 | 15.672 | 4.506 | -0.839 | 1.79 |
|  | 3 | 20.658 | 15.772 | 4.887 | -0.458 | 1.37 |

**B. *Il6***

| **Group** | **Mouse** | **Ct** | | **Δ Ct** | **ΔΔ Ct** | **Fold Change** |
| --- | --- | --- | --- | --- | --- | --- |
|  |  | ***Il6*** | ***Gapdh*** |  |  |  |
| Control | 1 | 26.438 | 15.787 | 10.652 | -0.404 | 1.32 |
|  | 2 | 26.735 | 15.665 | 11.070 | 0.014 | 0.99 |
|  | 3 | 27.329 | 15.884 | 11.445 | 0.390 | 0.76 |
| NMN | 1 | 27.919 | 15.506 | 12.413 | 1.358 | 0.39 |
|  | 2 | 28.372 | 15.577 | 12.796 | 1.740 | 0.30 |
|  | 3 | 27.346 | 15.670 | 11.676 | 0.621 | 0.65 |

**C. *Ppargc1a***

| **Group** | **Mouse** | **Ct** | | **Δ Ct** | **ΔΔ Ct** | **Fold Change** |
| --- | --- | --- | --- | --- | --- | --- |
|  |  | ***Ppargc1a*** | ***Gapdh*** |  |  |  |
| Control | 1 | 22.994 | 15.673 | 7.322 | 0.225 | 0.86 |
|  | 2 | 22.897 | 15.830 | 7.068 | -0.029 | 1.02 |
|  | 3 | 22.866 | 15.966 | 6.900 | -0.197 | 1.15 |
| NMN | 1 | 22.747 | 15.706 | 7.041 | -0.055 | 1.04 |
|  | 2 | 22.198 | 15.672 | 6.526 | -0.570 | 1.48 |
|  | 3 | 22.460 | 15.772 | 6.689 | -0.408 | 1.33 |

**D. SIRT3 and IL-6**

| **Group** | **Mouse** | **Protein** | **SIRT3** | | **IL-6** | |
| --- | --- | --- | --- | --- | --- | --- |
|  |  | **(mg/mL)** | **(pg/mL)** | **(pg/mg protein)** | **(pg/mL)** | **(pg/mg protein)** |
| Control | 1 | 3.16 | 67 | 21.23 | 3.37 | 1.07 |
|  | 2 | 2.70 | 19 | 7.03 | 2.45 | 0.91 |
|  | 3 | 3.25 | 67 | 20.61 | 2.22 | 0.68 |
|  | 4 | 1.42 | 9 | 6.33 | 1.07 | 0.75 |
|  | 5 | 2.70 | 75 | 27.76 | 2.68 | 0.99 |
|  | 6 | 2.91 | 67 | 23.05 | 6.58 | 2.26 |
| NMN | 1 | 2.22 | 107 | 48.22 | 3.37 | 1.52 |
|  | 2 | 2.87 | 75 | 26.17 | 1.53 | 0.53 |
|  | 3 | 2.65 | 79 | 29.80 | 1.30 | 0.49 |
|  | 4 | 4.26 | 204 | 47.91 | 1.53 | 0.36 |
|  | 5 | 2.98 | 71 | 23.83 | 1.76 | 0.59 |
|  | 6 | 2.80 | 87 | 31.03 | 3.37 | 1.20 |
