# Supplementary material for: Systemic nicotinamide mononucleotide administration to mitigate post-cardiac arrest brain injury in mice
Source: PLoS One. 2025 Oct 21;20(10):e0334608. doi: 10.1371/journal.pone.0334608 (PMC12539731; doi:10.1371/journal.pone.0334608)
Supplement: Attachment — Submitted filename: Response_to_Reviewers.docx [file pone.0334608.s006.docx]

**Manuscript Number: PONE-D-24-59975**

**Response to the reviewers**

Thank you for the constructive suggestions to improve our original manuscript. Our manuscript has benefited from these insightful suggestions. Our point-by-point response to each comment is written as plain text below the reviewer’s comments.

**Journal Requirements**

**1)** *When submitting your revision, we need you to address these additional requirements. Please ensure that your manuscript meets PLOS ONE's style requirements, including those for file naming. The PLOS ONE style templates can be found at https://journals.plos.org/plosone/s/file?id=wjVg/PLOSOne_formatting_sample_main_body.pdf and https://journals.plos.org/plosone/s/file?id=ba62/PLOSOne_formatting_sample_title_authors_affiliations.pdf*

**Response**: Thank you for pointing this out. The manuscript was revised to conform to the PLOS ONE format (e.g., title page, abstract, and main text).

**2)** *Thank you for your submission to PLOS ONE. We note that your study design may include death of a regulated animal as a likely outcome or planned experimental endpoint. At this time, we request that you please report additional details in your Methods section regarding animal care and use for the survival study, as per our editorial guidelines (http://journals.plos.org/plosone/s/submission-guidelines#loc-humane-endpoints). For easy reference, we have attached a checklist that may be relevant for your submission. Please complete all items on the checklist at the following link: http://journals.plos.org/plosone/s/file?id=bb1d/plos-one-humane-endpoints-checklist.docx Please upload the completed checklist as file type “Other” when resubmitting your manuscript. This document is for internal journal use only and will not be published if your article is accepted. We very much appreciate your attention to these requests and support of improved reporting standards in PLOS ONE submissions.*

**Response**: We appreciate your guidance. We revised the descriptions regarding animal care and humane endpoints in accordance with the PLOS ONE submission guidelines and attached a humane endpoints checklist.

**3)** *To comply with PLOS ONE submissions requirements, in your Methods section, please provide additional information regarding the experiments involving animals and ensure you have included details on (1) methods of sacrifice and (2) efforts to alleviate suffering.*

**Response**: Thank you for your guidance. We added additional descriptions about methods of sacrifice and efforts to alleviate suffering.

**4)** *Thank you for stating the following in the Competing Interests section: [I have read the journal's policy and the authors of this manuscript have the following competing interests: [J.Y. is listed as an inventor on patent applications related to NMN and adiponectin (US20180228824, JP2018131418A). The other authors declare no conflict of interest.]. Please confirm that this does not alter your adherence to all PLOS ONE policies on sharing data and materials, by including the following statement: ""This does not alter our adherence to PLOS ONE policies on sharing data and materials.” (as detailed online in our guide for authors http://journals.plos.org/plosone/s/competing-interests). If there are restrictions on sharing of data and/or materials, please state these. Please note that we cannot proceed with consideration of your article until this information has been declared. Please include your updated Competing Interests statement in your cover letter; we will change the online submission form on your behalf.*

**Response**: Thank you for pointing this out. We updated the competing interests statement in our cover letter, including the following statement: "This does not alter our adherence to PLOS ONE policies on sharing data and materials.”

**5)** *PLOS requires an ORCID iD for the corresponding author in Editorial Manager on papers submitted after December 6th, 2016. Please ensure that you have an ORCID iD and that it is validated in Editorial Manager. To do this, go to ‘Update my Information’ (in the upper left-hand corner of the main menu), and click on the Fetch/Validate link next to the ORCID field. This will take you to the ORCID site and allow you to create a new iD or authenticate a pre-existing iD in Editorial Manager.*

**Response**: Thank you for letting us know. We validated the ORCID iD in Editorial Manager.

**Reviewer 1**

*I read with interest the manuscript entitled « Systemic nicotinamide mononucleotide administration to mitigate post-cardiac arrest brain injury in mice » by Koichiro Homma et al. The authors provided robust data suggesting that nicotinamide mononucleotide administered intraperitoneally (first dose upon restoration of circulation) could improve neurological outcomes after KCl-induced cardiac arrest in mice. This is a first step towards potential application in human. They also tried to provide mechanistic insights by performing qPCR that revealed an increase in Sirtuin 3 expression and a decrease in IL-6 expression in brain of treated animals. Even though data on survival and brain damage are quite convincing, opening new opportunities to treat humans in the future, the mechanistic explorations are weak. I have the following comments:*

**Response**: We are grateful for your comments on our manuscript. Please see our specific responses below.

**1)** *The conclusion of the abstract stating that NMN restored mitochondrial biogenesis is not supported by the results. The authors must provide data on biogenesis to support this claim. The conclusion of the main article stating that “Nicotinamide mononucleotide showed these favorable effects at least in part through the restoration of the Sirt3/Ppargc1a pathway and reduction of Il-6 expression in the brain. » is not sufficiently supported by the results. There is an association, but no demonstration, of the mechanisms of protection.*

**Response:** We agree with this reviewer’s comment. Given that NMN has been previously reported to support mitochondrial function recovery [Neurochem Res. 2019;44: 2280–2287], we conducted an additional experiment to measure ATP levels in the brain. The results showed that NMN significantly increased ATP levels in the brain, suggesting the functional role of NMN in restoring mitochondrial function. A panel was added to Figure 2. Excerpt of the Figure and its legends are as follows:

**Fig 2. Post-ROSC administration of NMN increases NAD^+^ and ATP concentrations in the mouse brain.** (A) Brain NAD^+^ concentration is decreased after ROSC, and systemic NMN administration increases the brain NAD^+^ concentration 15 min and 2 h post-ROSC. (B) NMN increases the brain ATP concentration 2 h post-ROSC. Data are presented as means with standard errors. (A) One-way ANOVA with a post hoc 2-tailed Student’s t test, (B) Student’s t-test, *p < 0.05, **p < 0.01. ANOVA, analysis of variance; ATP, adenosine triphosphate; NAD^+^, nicotinamide adenine dinucleotide; NMN, nicotinamide mononucleotide; ROSC, return of spontaneous circulation.

As per your suggestion, we revised the wording to discuss the association between NMN and mitochondrial function and omitted claims of mechanisms. We have added and rewritten the text as follows:

In conclusion, systemic NMN administration after ROSC attenuates PCABI. The increased brain ATP levels and SIRT3 upregulation may suggest the usefulness of NMN for improving mitochondrial function and contributing to neuroprotection. NAD^+^ supplementation with NMN is a promising therapeutic approach against PCABI. (page 2, lines 44–47)

**Brain ATP assay** NMN treatment can support mitochondrial function as evidenced by higher brain tissue ATP levels [4]. Thus, ATP levels in brain tissues, including the cortex and hippocampus, at 2 h post-ROSC were measured using a luciferase ATP level assay kit (AMERIC-ATP(T) kit; Applied Medical Enzyme Research Institute Corporation, Tokushima, Japan) according to the manufacturer’s protocol. The brain tissues were homogenized with phenol-containing extraction reagent and centrifuged at 10,000 × g for 10 min, and the supernatants were collected. The supernatants were diluted 100-fold with ultrapure sterile water, and 10 µL of these diluted supernatants were mixed with 90 µL luciferase reagent. Then, bioluminescence was measured using a luminometer. Brain ATP levels (nmol/g tissue) in each sample were calculated from the calibration curve and the dilution ratio. (page 9, lines 173-181)

We measured brain ATP levels 2 h post-ROSC to assess the mitochondrial function in 10 mice assigned to either the NMN or control group (5 mice per group). NMN significantly increased the ATP levels in the brain 2 h post-ROSC (control: 203.6 ± 28.4 nmol/g tissue vs. NMN: 287.0 ± 21.9 nmol/g tissue, p = 0.048; Fig 2B). (page 13, lines 255–258)

Systemic NMN administration post-CA increases brain NAD^+^ and ATP levels, attenuates delayed neuronal injury 48 h post-CA, and improves neurological function and survival in a murine model of CA. These concurrently occurred with SIRT3 activation in the brain. (page 21, line 399–402)

**2)** *This study can be interpreted as a preclinical pilot study. The authors justified the choice of testing NMN, whereas the protective effects of NAD+ administration with niacin have been demonstrated because it is more tolerated and efficient to enter the cell than other drugs that can increase NAD+ levels. In this case, why did the authors not choose a route of administration (IV) that could be used in patients with CA?*

**Response:** Thank you for your question. Given that there were no previous reports of NMN administration in a model of cardiac arrest (CA), we adopted the intraperitoneal administration protocol that was used in a previous report of a murine model of cerebral infarction [Neurobiol Dis. 2016;95: 102–110]. Additionally, in this murine model of CA, we could not inject NMN intravenously immediately after achieving ROSC because continuous intravenous injection of epinephrine was required a few minutes after resuscitation. However, we agree with this reviewer’s comment that intravenous administration of NMN may have more advantages over intraperitoneal injection. Therefore, we have added a sentence discussing this point as a future direction in the limitations section.

Based on previous studies demonstrating that intraperitoneal NMN administration induces a rapid and sustained increase in NAD⁺ levels, the intraperitoneal route was selected over intravenous administration in this study [10–12]. (page4, lines 73–75)

Fourth, the protocol for NMN administration after CA (e.g., route [especially intravenously], timing, and dosage) needs to be optimized. (page 20, lines 389–390)

**3)** *NMN was administered immediately after ROSC. As shown by all RCT, it is not possible to administer a new drug within minutes after ROSC, which may explain the lack of translation of many promising drugs in clinical trials. It would be interesting to test whether delaying the administration of NMN is still protective in an additional group (to ensure that it can be tested in clinical trials). In any case, this point should be discussed from the perspective of translational medicine.*

**Response:** Thank you for constructive suggestion. We agree that our study design has less clinical feasibility, and testing the delayed administration of NMN should be considered for future studies. Although we believe that early NAD^+^ replenishment from NMN administration may confer better neuroprotection, a therapeutic time window may exist, as indicated by the gradual decrease in NAD^+^ concentrations during the first 15 minutes of ROSC (Fig 2A). A previous report supports the notion that delayed NMN administration at 30 min after reperfusion reduces neuronal damage in a murine model of cerebral infarction. We will evaluate the delayed administration of NMN in a future study. We have added the following text:

For future clinical translation, delayed NMN administration should be considered, as neuronal cells are protected by NMN administration even at 30 min after cerebral ischemia-reperfusion in the model of cerebral infarction [10]. (page 20, lines 390–392)

**4)** *For NAD levels (Experiment 1), can the authors provide data on sham animals? The question is whether NMN restores CA-induced depletion of NMN. Moreover, we tested whether NMN interfered with NAD+ measurement. Finally, how did the authors ascertain that NAD+ was measured in the brain cells and not in the extracellular fluid? (NMN may have modified blood NAD+ level without increasing brain cells NAD+ levels)*

**Response:** Thank you for these interesting questions. In response to your questions, we measured brain NAD^+^ concentrations in sham mice. The NAD^+^ concentrations in the control group 15 min post-ROSC tended to be lower than those in the sham group, but the difference did not reach significance. To clearly answer the question of whether NMN restored CA-induced depletion of NAD^+^, we also measured brain NAD^+^ levels in the control and NMN groups 2 h post-ROSC. The NAD^+^ concentrations were significantly lower in the control group than in the sham group, while those in the NMN group were significantly higher than in the control group. A figure panel was modified as follows:

**Fig 2. Post-ROSC administration of NMN increases NAD^+^ and ATP concentrations in the mouse brain.** (A) Brain NAD^+^ concentration is decreased after ROSC, and systemic NMN administration increases the brain NAD^+^ concentration 15 min and 2 h post-ROSC. (B) NMN increases the brain ATP concentration 2 h post-ROSC. Data are presented as means with standard errors. (A) One-way ANOVA with a post hoc 2-tailed Student’s t test, (B) Student’s t-test, *p < 0.05, **p < 0.01. ANOVA, analysis of variance; ATP, adenosine triphosphate; NAD^+^, nicotinamide adenine dinucleotide; NMN, nicotinamide mononucleotide; ROSC, return of spontaneous circulation.

We measured NAD^+^ concentrations using the NAD^+^ quantitation kit (EnzyChrom NAD/NADH Assay Kit [E2ND-100]; BioAassay Systems, Hayward, CA, USA), based on a lactate dehydrogenase (LDH) cycling reaction, in which the formed NADH reduced a formazan (MTT, 3-[4,5-dimethylthiazol-2-yl]-2,5-diphenyltetrazolium bromide) reagent. NMN cannot directly influence the LDH cycling and reduce the MTT reagent. Thus, we are confident that NMN did not interfere with NAD^+^ measurements in these experiments. NMN has been reported to increase NAD^+^ concentrations in hippocampal mitochondria [J Neurosci Res. 2019;97(8):975-990], suggesting that NMN increases intracellular NAD^+^ concentrations. Moreover, we found that NMN increased brain ATP content (Fig 2B). Considering that ATP is generated intracellularly, primarily by mitochondria, we consider that NMN increases intracellular NAD^+^ but not extracellular NAD^+^. We have made the following revisions:

At 15 min and 2 h after ROSC, brain tissues that included the cortex and hippocampus (≤ 20 mg) were harvested and snap frozen with liquid nitrogen. Sham-operated mice underwent the same procedure, including tracheal intubation and arteriovenous cannulation, but CA was not induced. Brain tissues were homogenized, and brain NAD^+^ concentrations were measured using the NAD^+^ quantitation kit (EnzyChrom NAD/NADH Assay Kit [E2ND-100]; BioAassay Systems, Hayward, CA, USA), a highly specific kit for NAD^+^/NADH, according to the manufacturer’s protocol. (pages 8–9, lines 166–171)

A total of 34 mice were used in this experiment. Four mice were used for the sham operation. A total of 2 mice did not achieve ROSC, and 28 mice were assigned to either the NMN or control group at 15 min and 2 h post-ROSC (n = 7 mice per group). The NAD^+^ concentration in the brain decreased gradually after ROSC and was significantly lower 2 h post-ROSC in control mice than in sham mice (sham: 169.7 ± 4.3 pmol/mg tissue vs. control [2 h post-ROSC]: 91.0 ± 8.7 pmol/mg tissue, difference: -78.7 [95% confidence interval (CI): -106.2 to -51.2], p = 0.0001; Fig 2A). Systemic NMN administration successfully increased the NAD^+^ concentration in the brain at 15 min post-ROSC (control: 141.4 ± 11.3 pmol/mg tissue vs. NMN: 185.6 ± 14.8 pmol/mg tissue, difference: 44.2 [95% CI, 3.7 to 84.6], p = 0.04) and 2 h post-ROSC (control: 91.0 ± 8.7 pmol/mg tissue vs. NMN: 231.2 ± 11.8 pmol/mg tissue, difference: 140.2 [95% CI, 108.3 to 172.0], p < 0.0001; Fig 2A). (pages 12–13, lines 247–255)

Brain NAD^+^ levels decreased post-ROSC, and intraperitoneal NMN administration increased brain NAD^+^ levels 15 min and 2 h post-ROSC, consistent with findings from a previous study on wild-type mice [11]. (page 18, lines 340–341)

**5)** *It is unclear whether the investigators who performed surgery and CPR were blinded to the experimental group. We understand that this was not the case in the limitations paragraph of the Discussion section. Please revise the methods to make them clearer to the reader.*

**Response:** Thank you for your suggestion. We added the following text:

This study was single-blinded. The investigators who performed surgery, CPR, and NMN or normal saline (NS) administration were aware of the treatment assignment. However, assessments of neurological function and histologic neuronal injury were performed by investigators blinded to treatment assignment. (page 7, lines 131–133)

**6)** *The dose of epinephrine administered during resuscitation has not been reported in the Results section. Therefore, it is important data to provide to ensure that the two groups of animals are comparable.*

**Response:** Thank you for this comment. Continuous epinephrine administration was started 10 min after cardiac arrest and discontinued when MAP of 70 mmHg was maintained. There were no significant differences in the epinephrine dose between the two groups. We added the following data and text.

**Table 1. Comparison of baseline characteristics, resuscitation details, and vital signs between the two groups of mice.**

|  | **Control group (n = 18)** | **NMN group (n = 18)** | **p value** |
| --- | --- | --- | --- |
| **Epinephrine dose (μg)** | 7.1 (6.0–8.1) | 6.3 (6.0–7.7) | 0.57 |

The baseline characteristics, CPR time, and epinephrine dose were similar between the two groups (Table 1). (page 14, lines 270–271)

**7)** *The authors measured SIRT3 expression as well as IL-6 and found an increase and a decrease, respectively. However, it is unclear why they did not measure SIRT3 levels and activity IL-6 levels. Moreover, the authors did not provide data directly linking NMN administration with the abovementioned results. Finally, the authors declared that “Nicotinamide mononucleotide improved Sirt3/Ppargc1a expression » (Line 268) which is not completely true as Ppargc1a expression was not significantly different between the 2 experimental groups.*

**Response:** Thank you for the comment. As suggested, we measured the protein levels of SIRT3 and IL-6 in the brain. We found that NMN upregulated *Sirt3* expression and increased the protein levels of SIRT3. IL-6 protein levels in the brain showed a trend toward a decrease with NMN, but the difference did not reach significance. This may reflect the large inter-sample variations, as well as the time lag between RNA to protein transcription. We agree with this reviewer’s comment that we do not have sufficient data to support the direct mechanisms by which NMN increased SIRT3 levels. Therefore, in our revised manuscript, we removed the mechanistic claims in the Results and only discussed the potential mechanisms in the Discussion. The changes are as follows:

**Fig 5. NMN increases SIRT3 expression at both mRNA and protein levels in the brain 6 h after cardiac arrest.** Relative RNA expressions to control in hippocampi are measured 6 h after cardiac arrest (n = 3 per group). (A) *Sirt3*, (B) *Il6*, and (C) *Ppargc1a*. Hippocampal protein levels of SIRT3 and IL-6 are also measured 6 h after cardiac arrest (n = 6 per group). (D) SIRT3, and (E) IL-6. Data are presented as means with standard errors or medians with interquartile ranges. Student’s t-test, *p < 0.05. *Il6*, interleukin-6 (gene); IL-6, interleukin-6 (protein); NMN, nicotinamide mononucleotide; n.s., not significant; *Ppargc1a*, peroxisome proliferator-activated receptor gamma coactivator 1-alpha; RNA, ribonucleic acid; *Sirt3*, sirtuin-3 (gene); SIRT3, sirtuin-3 (protein).

**Brain ELISA analysis** The protein levels of SIRT3 and IL-6 in the brain were measured using ELISA kits (SEE913Mu for SIRT3; Cloud-Clone Corporation, Katy, TX, USA. M6000B-1 for IL-6; R&D Systems, Minneapolis, MN, USA) according to the manufacturer’s instructions. Briefly, the hippocampi were homogenized in the radio-immunoprecipitation assay buffer containing 50 mM Tris-HCl (pH 8.0), 150 mM sodium chloride, 0.5 w/v% sodium deoxycholate, 0.1 w/v% sodium dodecyl sulfate, and 1.0 w/v% NP-40 substitute (RIPA Buffer, FUJIFILM, Osaka, Japan) with a protease inhibitor cocktail containing 1 mM AEBSF, 800 nM aprotinin, 50 μM bestatin, 15 μM E64, 20 μM leupeptin, and 10 μM pepstatin A (Halt™ Protease Inhibitor Cocktail, Thermo Fisher Scientific, Rockford, IL, USA). Homogenates were centrifuged at 10,000 × *g* for 5 min at 4°C, and the supernatant was then collected. Bicinchoninic acid assay of the supernatant was performed to quantify total protein concentrations (Pierce™ BCA Protein Assay Kits, Thermo Fisher Scientific). The levels of SIRT3 and IL-6 in the supernatant were measured using ELISA kits and normalized to the total protein concentration of the sample. (pages 11–12, lines 221–232)

**NMN increased SIRT3 expression in the hippocampus** We measured the mRNA expressions of *Sirt3*, *Il6*, and *Ppargc1a* (the murine gene of PCG-1α gene regulating SIRT3 expression) (Fig 5A–C) and the protein levels of SIRT3 and IL-6 (Figs 5D and 5E) in the hippocampi of the control and NMN groups at 6 h post-CA. NMN significantly upregulated relative *Sirt3* expression (control: 1.0 ± 0.1 vs. NMN: 1.5 ± 0.1, difference: 0.5 [95% CI, 0.04 to 1.0], p = 0.04; Fig 5A) and increased the protein levels of SIRT3 (control: 17.7 ± 3.6 vs. NMN: 34.5 ± 4.4 pg/mg tissue, difference: 16.8 [95% CI, 4.1 to 29.6], p = 0.01; Fig 5D). Similarly, NMN significantly downregulated relative *Il6* expression (control: 1.0 ± 0.2 vs. NMN: 0.4 ± 0.1, difference: -0.6 [95% CI, -1.1 to -0.03], p = 0.04; Fig 5B). IL-6 levels in the brain showed a trend toward decrease in the NMN group, but the difference did not reach significance (control: 0.95 pg/mg tissue [IQR: 0.73–1.37] vs. NMN: 0.56 pg/mg tissue [IQR: 0.46–1.28], p = 0.24; Fig 5E). The NMN group also showed a trend toward higher *Ppargc1a* expression, although the difference did not reach significance (control: 1.0 ± 0.1 vs. NMN: 1.3 ± 0.1, difference: 0.3 [95% CI, -0.2 to 0.7], p = 0.15; Fig 5C). The qPCR cycle threshold values and specific protein levels of individual mice are shown in S4 Table. In summary, NMN increased SIRT3 in the hippocampus early after CA. (page 16–17, lines 312–325)

This study revealed that systemic NMN administration post-ROSC increased NAD^+^ and ATP levels in the brain and improved NFS, survival, and histological neuronal injury in a murine model of CA. This concurrently occurred with SIRT3 activation in the brain. (page 18, lines 336–338)

**8)** *Introduction*

*- Reference 3, published in 2013, is not really « recent » as suggested by the authors in the introduction (line 46, page 3);*

**Response:** Thank you for the comment. We revised the texts as follows:

Nicotinamide adenine dinucleotide (NAD^+^) depletion after ischemia-reperfusion, including post-CA, contributes to neuronal injury [2,3]. (page 3, lines 55–56)

*- Please, clarify how CA was induced in the abstract as the KCl-induced CA is not a usual cause of CA -*

**Response:** KCl-induced cardiac arrest is the commonly used experimental model in mice. To improve clarity, we made the following amendment to the abstract:

In adult male mice (C57BL/6NCrSlc, 10–15 weeks old), 10-min CA was induced by intravenous potassium chloride injection followed by cardiopulmonary resuscitation. (page 2, lines 35–36)

*- Justify the intraperitoneal route for the adminstration of NMN (rather than intravenous)*

**Response:** This is the same as your comment # 2. Please see our response above.

**9)** *Results*

*- There are elements of methods or discussion in the Results: See, for example lines 172-174 (Mitochondrial injury occurs rapidly after ROSC [24], making timely transport of NMN across the blood– brain barrier crucial for preventing and treating PCABI. A previous study reported that NMN rapidly increases brain NAD+ levels [11]). Please revise the entire section accordingly.*

**Response:** Thank you. We revised the Results section per your suggestion and moved elements to the Methods and Discussion. Please refer to the revised text.

*- Line 180: these data are already available in Figure 2*

**Response:** Thank you. These data were removed from the text to avoid duplication.

*- Line 193 : « Despite a longer CPR time in the NMN group, MAP showed an elevated trend in the NMN group at 30 min after ROSC. » Given the results in Table 1, there was no difference between the two groups regarding these parameters. Please revise.*

*- Please provide body temperature at baseline in Table 1*

**Response:** Thank you for pointing this out. We have revised the text as follows:

There were also no significant differences in vital signs at 30 min after ROSC between groups. (page 14, lines 271–272)

The cardiovascular effects of NMN were unclear in our study. There was no significant difference in MAP between the two groups at 30 min post-ROSC (Table 1). (pages 18–19, lines 353–355)

**Table 1. Comparison of baseline characteristics, resuscitation details, and vital signs between the two groups of mice.**

|  | **Control group (n = 18)** | **NMN group (n = 18)** | **p value^a^** |
| --- | --- | --- | --- |
| **Physiological variables** |  |  |  |
| **Baseline** |  |  |  |
| **BT (℃)** | 35.9 ± 0.2 | 35.5 ± 0.2 | 0.15 |

**10)** *Discussion:*

*- Line 280 Nicotinamide mononucleotide should be changed in « NMN ». See also line 320.*

**Response:** Thank you. We have ensured that this was changed throughout the text.

*- Lines 293-296. See the last point on the major limitations. The same is true for the next paragraph.*

**Response:** Thank you for pointing this out. We have revised the texts as follows:

NMN administration improved 7-day survival and neurological function 48 h post-CA. (page 18, line 350)

Systemic NMN administration post-CA increases brain NAD^+^ and ATP levels, attenuates delayed neuronal injury 48 h post-CA, and improves neurological function and survival in a murine model of CA. These concurrently occurred with SIRT3 activation in the brain. Systemic NMN administration is a promising therapeutic approach for replenishing NAD^+^ in the brain and improving PCABI outcomes. (page 21, lines 399–402)

NMN also significantly increased ATP levels in the brain post-CA (Fig 2B). ATP levels may reflect mitochondrial function because mitochondria are the main organelles responsible for the ATP production [45]. Cellular ATP levels can decrease after ischemia-reperfusion due to a delayed perturbation of mitochondrial function [46]. Therefore, the increased ATP levels in the NMN group may suggest improved mitochondrial function. Moreover, we found that NMN administration increased post-CA SIRT3 levels in the brain (Fig 5). NMN preserves post-ischemic mitochondrial function via SIRT3-dependent mechanisms, including mitochondrial protein deacetylation, normalization of ROS production, and inhibition of mitochondrial fragmentation [12]. Our findings collectively suggest that NMN possibly improves post-CA mitochondrial function via SIRT3 activation. The direct mechanisms through which NMN improves mitochondrial function warrant further mechanistic studies. PGC-1α is a marker of mitochondrial biogenesis and interacts with SIRT3. The current study measured the expression of *Ppargc1a* in the brain post-CA and found no significant difference between groups (Fig 5C). This may be attributable to SIRT3 being regulated by multiple pathways in addition to PGC-1α. (page 19–20, lines 364–375)

NMN exerts anti-inflammatory effects, including the attenuation of IL-6 production in models of intracerebral hemorrhage, traumatic brain injury and sepsis-associated encephalopathy [17,25,47]. Increased systemic IL-6 levels are associated with increased mortality and poor neurological prognosis following CA [48–50]. Therefore, we next investigated whether NMN improvement of post-CA neuronal injury was mediated by the attenuation of IL-6 levels. The NMN group showed significantly lower *Il6* expression post-CA (Fig 5B), whereas there was no significant difference in the protein levels of IL-6 between groups (Fig 5E). This may be due to the suboptimal timing of the protein measurement in detecting the difference, as protein synthesis from mRNA requires time. (page 20, lines 376–383)

**Reviewer 2**

*Kaito et al. investigated the effects of nicotinamide mononucleotide (NMN) administration on neurological outcomes in a mouse model of cardiac arrest. NMN supplementation aims to replenish NAD+ levels, which become depleted following ischemia. The authors conducted a comprehensive study comprising four successive experimental approaches to assess cerebral NAD+ concentrations, neurological dysfunction and survival, brain lesions, and transcript levels of cerebral inflammatory markers. This is an elegant in vivo study, with well-designed experiments that provide valuable insights into the topic. The manuscript is well written, and the data are clearly presented. Notably, all individual data points are available in the supplementary materials, ensuring transparency and reproducibility. I have only minor comments:*

**Response:** We appreciate your comments on the manuscript. Our detailed responses to each comment are provided below.

**1)** *Page 5, Line 90: "Sample size for the outcomes..." – Could the authors clarify the initial hypothesis regarding the expected difference? Specifically, please provide the anticipated effect size along with the expected mean and standard deviation (SD) under control conditions.*

**Response:** Thank you for bringing this to our attention. Based on previous reports, we determined the effect size of NMN as follows:

The target sample sizes for the outcomes were calculated to provide 80% power to detect differences based on previous reports and our preliminary experiments, with a two-sided alpha level of 0.05. For the brain NAD^+^ assay, 32 mice were required. Particularly, a sample size of 14 mice was estimated to detect a mean difference of 20 pmol/mg tissue (standard deviation [SD]: 12 pmol/mg tissue) in brain NAD^+^ levels between groups at 15 min post-ROSC (control: 160 pmol/mg tissue vs. NMN: 180 pmol/mg tissue) [3,11,17]. A sample size of 14 mice was estimated to detect a mean difference of 60 pmol/mg tissue (SD: 35 pmol/mg tissue) in brain NAD^+^ levels between groups at 2 h post-ROSC (control: 140 pmol/mg tissue vs. NMN 200 pmol/mg tissue) [3,10,11,17]. Finally, a sample size of 4 sham-operated mice was estimated to detect a mean difference of 50 pmol/mg tissue (SD: 22 pmol/mg tissue) in brain NAD^+^ levels between groups (sham: 170 pmol/mg tissue vs. control [2 h post-ROSC]: 120 pmol/mg tissue) [3,10,11,17]. For the brain ATP assay, a sample size of 10 mice was estimated to detect a mean difference of 150 nmol/g tissue (SD: 70 nmol/g tissue) in brain ATP levels between groups at 2 h post-ROSC (control: 250 nmol/g tissue vs. NMN: 400 nmol/g tissue) [5,11,18–20]. (pages 5–6, lines 100–111)

For the neurological function and survival analysis, 36 mice were required (18 mice per group). Particularly, a sample size of 20 mice was estimated to detect a mean difference of 4 (SD: 3) in NFS between groups at 48 h post-CA (control: 7 vs. NMN: 11) [3,10,21], and a sample size of 36 mice was estimated to detect an absolute 40% difference (control: 20% vs. NMN: 60%) in the survival rate [5,9,22]. For the histological analysis, 20 mice were required. A sample size of 10 mice was estimated to detect a mean difference of 7% (SD: 3%) in Fluoro-Jade C (FJC)-positive areas between groups at 48 h post-ROSC (control: 10% vs. NMN: 3%) [3,10,23]. To follow the time course, the same number of mice was experimented at 24 h post-ROSC. (page 6, lines 112–118)

For the brain RNA analysis, 6 mice were required. We estimated a sample size of 6 mice to detect a mean difference of 0.5 (SD: 0.1) in relative *Sirt3* expression between groups (control: 0.5 vs. NMN: 1.0) [5,20,24]. A sample size of 6 mice was also estimated to detect a mean difference of 0.5 (SD: 0.1) in relative *Il6* expression between groups (control: 1.0 vs. NMN: 0.5) [23,25]. For the brain ELISA analysis, 12 mice were required. We estimated a sample size of 12 mice to detect a mean difference of 10 pg/mg tissue (SD: 10 pg/mg tissue) in SIRT3 protein levels between groups (control: 20 pg/mg tissue vs. NMN: 40 pg/mg tissue) [5,20,24,26]. A sample size of 12 mice was also estimated to detect a mean difference of 1.0 pg/mg (SD: 0.5 pg/mg) in IL-6 protein levels between groups (control: 2.0 pg/mg protein vs. 1.0 pg/mg protein) [23,25,27]. (page 6, lines 119–126)

**2)** *Page 6, Line 120: The route, timing, dosage, and volume of NMN administration should be explicitly stated in this section rather than solely in the Results.*

**Response:** Thank you for this comment. We revised the text as follows to improve clarity.

NMN at a dose of 60 mg/kg dissolved in 200 µL NS was immediately administered post-ROSC via intraperitoneal injection as described in a previous murine model of cerebral ischemia [10]. The control group received the same amount of NS via intraperitoneal injection. The same administration of NMN or NS was repeated 24 and 48 h post-CA. NMN was purchased from Oriental Yeast Co. Ltd. (Tokyo, Japan). (page 8, lines 157–160)

**3)** *Page 9, Lines 172–175: This paragraph should be moved to the Introduction of the Methods section, as it is not a result but rather methodological background.*

**Response:** Thank you. We moved the paragraph as suggested.

**4)** *Figure 1: The timing of brain sample collection in Experiments 1 and 4 is unclear and should be explicitly indicated in the figure.*

**Response:** Thank you for this important comment. We added the timing of brain sample collection in Fig 1.

**Fig 1. Schematic summary of the experimental protocols.** Mice are assigned to the NMN group (NMN 60 mg/kg) or the control group (equivalent volume of NS). NMN or NS is administered at 0, 24, and 48 h after cardiac arrest. The following 4 experiments are conducted: experiment 1: brain NAD^+^ and ATP assays; experiment 2: neurological function and survival analysis; experiment 3: histological analysis of hippocampi; and experiment 4: brain RNA and ELISA analyses. ATP, adenosine triphosphate; BT, body temperature; CA, cardiac arrest; CPR, cardiopulmonary resuscitation; ELISA, enzyme-linked immunosorbent assay; Exp, experiment; KCL, potassium chloride; NAD^+^, nicotinamide adenine dinucleotide; NFS, neurological function score; NMN, nicotinamide mononucleotide; NS, normal saline; RNA, ribonucleic acid; ROSC, return of spontaneous circulation.

**5)** *Figure 3: The Methods section states that animals that died were excluded from the neurological dysfunction analysis. However, in the figure, several scores appear at 12. Could the authors clarify whether these scores correspond to surviving animals? Additionally, since many scoring systems use death as the maximum score, it would be helpful to specify how mortality is represented in the figure. I believe that 12 corresponds to dead animals and that these animals just did not participate to the statistical analysis but this is counfounding if mentionned in the figure. If it is right, the authors could invert left and right panel and presents only alive animal in the NDS panel.*

**Response:** Thank you for the comment. The established Neurological Function Score (NFS) was used to assess neurological function post-CA in mice [Circulation. 2004;109: 2786–2791]. Unlike other scoring systems, such as NDS, NFS comprises a score ranging from 0 (death) to 12 (good neurologic function), with higher scores indicating better neurological function. The reviewer is right. We compared the neurological function of surviving mice, and dead mice were excluded in these comparisons. Although it seems that it is common to retain dead mice in the figure panel but exclude them from the analysis, we removed the dead mice as suggested to improve clarity. The revised figure panel is pasted below for your reference.

**Fig 3. NMN improves neurological function and survival after cardiac arrest in mice.** (A) Mouse neurological function score 24 and 48 h after cardiac arrest (n = 18 per group, dead mice are excluded from the statistical analysis). (B) Mouse survival 7 days after cardiac arrest (n = 18 per group). (A) Mann–Whitney U test, (B) log-rank test, *p < 0.05. CA, cardiac arrest; NMN, nicotinamide mononucleotide; n.s., not significant.
