# Supplementary material for: Systemic nicotinamide mononucleotide administration to mitigate post-cardiac arrest brain injury in mice
Source: PLoS One. 2025 Oct 21;20(10):e0334608. doi: 10.1371/journal.pone.0334608 (PMC12539731; doi:10.1371/journal.pone.0334608)
Supplement: Attachment — Submitted filename: Response_to_Reviewers_auresp_2.docx [file pone.0334608.s007.docx]

**Manuscript Number: PONE-D-24-59975R1**

**Response to the reviewers**

We sincerely thank the reviewers for their thoughtful and constructive comments, which have helped us improve the clarity and quality of our work. Our point-by-point responses to each comment are provided below.

**Journal Requirements**

*If the reviewer comments include a recommendation to cite specific previously published works, please review and evaluate these publications to determine whether they are relevant and should be cited. There is no requirement to cite these works unless the editor has indicated otherwise.*

**Response**: We appreciate this suggestion. Please see our specific response to Reviewer #1 regarding the cited work. We added a paragraph to the discussion and cited the referenced paper (Abella, Circulation. 2004;109:2786–2791).

**Reviewer #1**

*R2 - Systemic nicotinamide mononucleotide administration to mitigate post-cardiac arrest brain injury in mice » by Koichiro Homma et al. I thank the authors for their thorough responses to my previous comments and for the significant improvements made to the manuscript. The revisions have strengthened the study. Nevertheless, I still have one substantive concern regarding the analysis of the neurological function score, which is central to the manuscript’s main conclusion. In response to reviewer 2, the authors modified Figure 3. However, in Figure 3A, neurological scores are reported as significantly higher at 48 hours post-ROSC, while it seems that animals who died were excluded from this analysis. This approach is problematic, since the scoring system assigns a value of 0 to deceased animals. When recalculating the statistics using the individual scores provided in Table S2, and including the deceased animals (as described in the original method by Neumar et al., Circulation 2004; 109:2786–2791), the difference in neurological outcome is no longer statistically significant (p = 0.08). Therefore, the manuscript’s principal conclusion—that nicotinamide improves neurological function—requires revision. I recommend that the authors present the analysis transparently, including all animals in accordance with the validated scoring system, and adjust both the results and their interpretation accordingly. If the authors choose to retain the current analysis, they should provide a clear justification and cite supporting validation from the literature, and describe the causes of death of the excluded animals as well as the humane endpoints if death did not occur spontaneously.*

**Response**: We appreciate your comments on our revised manuscript. As you pointed out, the original publication of the mouse cardiac arrest (CA) model (Abella, Circulation 2004) included deceased mice when assessing neurological function scores (NFS). However, in that work, a large proportion of mice had died by 18 and 72 h post-CA, and so they also analyzed NFS among only survivors, but no differences were observed in NFS. The authors themselves acknowledged this limitation, stating in the results section that "our ability to make a valid comparison is limited."

Including dead mice as having a score of zero in the statistical analysis artificially lowers the median NFS and can obscure the differences between experimental groups, especially when survival rates differ due to treatment effects. Conversely, an approach that assesses NFS only among surviving mice accurately reflects the neurofunctional outcomes of surviving animals, without conflating neurological impairment with mortality. Consequently, an approach of assessing NFS among the surviving mice has been validated in multiple studies and has become the prevailing method in recent years, as evidenced by several articles published in prominent journals (Dezfulian, Circulation 2009; Minamishima, Circulation 2009; Hayashida, Circulation 2019; Tamura, Sci Transl Med 2024). Based on these practices, our experimental design adopted assessing NFS among surviving mice and assessing survival separately.

We presumed that the notion that dead mice should preferably be retained in the NFS analysis is based on the assumption that all early deaths occur after brain injury. Although acute mortality is thought to be primarily due to post-CA brain injury, especially within the first 72 h post-CA, mortality from cardiovascular instability or multiorgan failure can also occur (Sandroni, Intensive Care Med 2021). In animal models, the exact cause of death is often unclear even upon autopsy, and not all deaths can be attributed to brain injury. In our experiments, no mice were sacrificed for the humane endpoint related to brain injury; therefore, analyzing deceased mice as having an NFS of 0 could introduce bias, as deaths due to non-neurological causes would be incorrectly classified as poor neurological outcomes. Based on these biological reasons and previous publications, we respectfully disagree with the reviewer’s comment and chose to analyze NFS only among surviving mice and report survival separately.

The reviewer made a valid point that the data must be communicated transparently. Therefore, we added dead mice to the figure panel (Fig 3A). The figure panel, figure legend, and excerpt of modified text are pasted below for your convenience.

**Fig 3. NMN improves neurological function and survival after cardiac arrest in mice.** (A) Neurological function scores (NFS) at 24 and 48 h after cardiac arrest (n = 18 per group; NFS analyzed only in surviving mice).

To highlight that the analyses of NFS were prespecified and performed solely on surviving mice, and that no mice were sacrificed due to humane endpoints, we revised the manuscript as follows:

At 48 h post-CA, surviving mice in the NMN group exhibited significantly higher NFS (control: 8 [IQR: 4–12] vs. NMN: 12 [IQR: 9–12], p = 0.03) and less severe hippocampal neuronal injury compared with controls. (page 2, lines 41–42)

The primary outcome measure of the neurological function and survival analysis was the neurological function score (NFS) at 48 h post-CA among surviving mice, while the secondary outcomes were the NFS 24 h post-CA among surviving mice and 7-day survival. (page 4–5, lines 87–90)

Particularly, a sample size of 20 mice was estimated to detect a mean difference of 4 (SD: 3) in NFS between groups at 48 h post-CA among surviving mice (control: 7 vs. NMN: 11) [3,10,21] (page 6, lines 113–115)

The prespecified statistical analysis of NFS at 24 and 48 h post-CA was performed among surviving mice only, with dead mice excluded from the comparisons in accordance with prior CA model studies [21, 28, 30]. (page 12, lines 241–243)

At 24 h post-CA, surviving mice in the NMN group showed a trend toward improved NFS (control: 7 [IQR: 6–11] vs. NMN: 12 [IQR: 5–12], p = 0.35; Fig 3A). (page 15, lines 286–287)

Furthermore, repeated NMN administration significantly improved NFS at 48 h post-CA in surviving mice (control: 8 [IQR: 4–12] vs. NMN: 12 [IQR: 9–12], p = 0.03; Fig 3A). (page 15, lines 289–290)

No mice were sacrificed due to the humane endpoint. (page 15, line 292)

To address the concern raised by this reviewer, we added a paragraph to the Discussion section as follows:

NMN administration improved neurological function at 48 h post-CA among surviving mice (Fig 3A). In this study, we analyzed NFS only in surviving mice, as this approach more accurately reflects the neurological outcomes without conflating neurological impairment with mortality. This approach of evaluating NFS exclusively in surviving mice after CA has been validated in multiple studies and has become the prevailing standard in recent years [21, 28, 30]. The original publication of the mouse CA model assessed NFS by including deceased mice [43]. However, in that study, a large number of mice had died by 18 and 72 h post-CA, and no difference in NFS was observed when analyzing only the surviving mice. Although acute mortality is often attributed to PCABI, deaths from cardiovascular instability or multiorgan failure can also occur [1]. In animal models, the exact cause of death is often unclear even upon autopsy, and not all deaths can be attributed to brain injury. In our experiments, no mice were sacrificed due to the humane endpoint related to brain injury. Therefore, including deceased mice in the analysis of neurological outcomes with an NFS of 0 could introduce bias, as deaths from non-neurological causes would also be classified as poor neurological outcomes. (page 18–19, lines 354–365)

**Reviewer #2**

*Thanks for the revision. No additional comment.*

**Response:** We appreciate you taking the time to review our revised manuscript.
